# Predicting the immediate impact of national lockdown on neovascular age-related macular degeneration and associated visual morbidity: an INSIGHT Health Data Research Hub for Eye Health report

**DOI:** 10.1136/bjophthalmol-2021-319383

**Published:** 2021-09-13

**Authors:** Susan P Mollan, Dun Jack Fu, Ching-Yi Chuo, Jacqueline G Gannon, Wen Hwa Lee, J Jill Hopkins, Cian Hughes, Alastair K Denniston, Pearse A Keane, Ronald Cantrell

**Affiliations:** 1 Ophthalmology, University Hospitals Birmingham, Birmingham, UK; 2 Research Centre, Moorfields Eye Hospital NHS Foundation Trust, London, UK; 3 Genentech Inc, South San Francisco, California, USA; 4 Action Against Age-Related Macular Degeneration, Oxford, UK; 5 Google Inc, Mountain View, California, USA; 6 Department of Ophthalmology, University Hospitals Birmingham NHSFT, Birmingham, UK; 7 Medical Retina, Moorfields Eye Hospital NHS Foundation Trust, London, UK

**Keywords:** neovascularisation, COVID-19, clinical trial

## Abstract

**Objective:**

Predicting the impact of neovascular age-related macular degeneration (nAMD) service disruption on visual outcomes following national lockdown in the UK to contain SARS-CoV-2.

**Methods and analysis:**

This retrospective cohort study includes deidentified data from 2229 UK patients from the INSIGHT Health Data Research digital hub. We forecasted the number of treatment-naïve nAMD patients requiring anti-vascular endothelial growth factor (anti-VEGF) initiation during UK lockdown (16 March 2020 through 31 July 2020) at Moorfields Eye Hospital (MEH) and University Hospitals Birmingham (UHB). Best-measured visual acuity (VA) changes without anti-VEGF therapy were predicted using post hoc analysis of Minimally Classic/Occult Trial of the Anti-VEGF Antibody Ranibizumab in the Treatment of Neovascular AMD trial sham-control arm data (n=238).

**Results:**

At our centres, 376 patients were predicted to require anti-VEGF initiation during lockdown (MEH: 325; UHB: 51). Without treatment, mean VA was projected to decline after 12 months. The proportion of eyes in the MEH cohort predicted to maintain the key positive visual outcome of ≥70 ETDRS letters (Snellen equivalent 6/12) fell from 25.5% at baseline to 5.8% at 12 months (UHB: 9.8%–7.8%). Similarly, eyes with VA <25 ETDRS letters (6/96) were predicted to increase from 4.3% to 14.2% at MEH (UHB: 5.9%–7.8%) after 12 months without treatment.

**Conclusions:**

Here, we demonstrate how combining data from a recently founded national digital health data repository with historical industry-funded clinical trial data can enhance predictive modelling in nAMD. The demonstrated detrimental effects of prolonged treatment delay should incentivise healthcare providers to support nAMD patients accessing care in safe environments.

**Trial registration number:**

NCT00056836.

**Key messages**

**What is already known about this subject?**

- Enforced national lockdowns to limit the spread of SARS-CoV-2 across the world have led to some restrictions in accessing non-essential primary care and hospital services.
- Delays in neovascular age-related macular degeneration (nAMD) diagnosis and treatment were subsequently reported, but the impact of disruption in ophthalmic services on treatment-naïve patients with nAMD requiring anti-vascular endothelial growth factor therapy is unknown.

**What are the new findings?**

- In this retrospective cohort study, using models based on data from the INSIGHT Health Data Research digital hub and the landmark Minimally Classic/Occult Trial of the Anti-VEGF Antibody Ranibizumab in the Treatment of Neovascular AMD trial, 376 nAMD patients were forecasted to have required treatment initiation at two major clinical centres during the UK national lockdown.
- Delays in their treatment were projected to have clinically relevant effects on their visual acuity.

**How might these results change the focus of research or clinical practice?**

- Efficient resumption of ophthalmic services following national lockdown is crucial to maximise positive visual outcomes for nAMD patients.

## Introduction

Neovascular age-related macular degeneration (nAMD) is the leading cause of irreversible blindness and vision impairment in the elderly population of high-income countries such as the UK.^1 2^ Visual impairment caused by nAMD can lead to devastating decrements in patients’ quality of life and poses a growing healthcare challenge, with the number of patients affected estimated to increase by >30% in a decade.^3–7^ Critically, visual prognosis can be substantially improved with intravitreal injection of vascular endothelial growth factors inhibitors (anti-VEGF).^8^ ^9^ It has been demonstrated that timely treatment initiation and active surveillance are essential for preservation of vision in patients with nAMD.^10^

The emergence and rapid spread of the new and highly contagious SARS-CoV-2, the pathogen causing COVID-19, has challenged healthcare systems across the globe. The exponential rise in severe COVID-19 cases has mandated a pandemic response requiring a redirection of healthcare resources to intensive care units, and measures to protect uninfected patients and healthcare providers from infection. These efforts have effectively shut down health services that are not critical for life. During the first enforced UK national lockdown, access to primary care services, optometric and hospital eye services was strictly limited to absolute emergencies. This led to delay of new-onset nAMD diagnosis and continuation of care for those already on the nAMD treatment pathway.^11–13^

The impact of this disruption on ophthalmic services and consequently on visual outcomes remains undetermined.^14^ Accurate forecasting of the disruption’s impact on ophthalmic services may have on nAMD treatment outcomes is important on several levels to maintain a high quality of care. Good predictions are required to inform triage and treatment strategies once services resume. Furthermore, they may support patient-level prognosis and shared decision-making. Lastly, forecasting visual outcomes is relevant in underpinning public health messaging and patient outreach services that ensure all patients are empowered to seek necessary care.

The INSIGHT Health Data Research Hub for Eye Health in the UK is uniquely positioned to explore such national health trends. As a national data collection initiative, INSIGHT aims to maximise the benefits and impact of historical, deidentified, patient-level UK National Health Service (NHS) hospital admission and electronic health record data by establishing formal collaborations between NHS Trusts, academia, patients, charities and industry. To this end, data from University Hospitals Birmingham (UHB) and Moorfields Eye Hospital NHS Foundation Trusts (MEH) were assembled to describe the period trends for patients initiating anti-VEGF therapy for nAMD and to forecast the number and characteristics of patients needing to initiate anti-VEGF for nAMD during the national lockdown.

To extrapolate the impact of treatment delay, the landmark Minimally Classic/Occult Trial of the Anti-VEGF Antibody Ranibizumab in the Treatment of Neovascular AMD trial (MARINA)^8^ sham-control arm data were used to model visual outcomes of the patients projected to have their treatment delayed by the COVID-19 lockdown. The MARINA trial was a phase III, randomised, multicentre, double-masked, sham-controlled study enrolling participants diagnosed with minimally classic nAMD or occult with no classic nAMD. Importantly, the MARINA trial is the last published study to include a sham-control arm, as anti-VEGF treatment became the gold standard of care for active nAMD thereafter.^15^

## Methods

### Study design

This retrospective cohort study includes treatment-naïve patients with nAMD initiating anti-VEGF therapy during the pre-COVID-19 era (1 January 2018 through 15 March 2020) at UHB and MEH. These data were used to forecast nAMD patients requiring treatment initiation in a specific time window of national lockdown where non-essential ophthalmic services were interrupted as part of the pandemic response in the UK (16 March 2020 through 31 July 2020). Visual outcomes were modelled based on patients in the control arm of the MARINA trial.^8^

### Data source

Electronic health record data gathered as part of routine clinical care at UHB and MEH between 1 January 2018 and 15 March 2020 were obtained via the INSIGHT collaborative Health Data Research Hub for Eye Health—a formal collaboration between UHB, MEH, Roche, Google, Action Against AMD and the University of Birmingham. Demographic characteristics included sex, ethnicity, age at diagnosis and smoking status. To ensure deidentification of patient data, age and ethnicity were categorised. Best-measured visual acuity (VA) with habitual correction or pinhole as part of clinical examination using ETDRS charts was recorded. For any cases where VA was reported in Snellen annotation, approximate ETDRS was extrapolated and taken forward for analysis.^16^

### Participants

Treatment-naïve nAMD patients requiring anti-VEGF therapy initiation at MEH and UHB between 1 January 2018 and 15 March 2020. Study eye was designated as the first eye diagnosed with nAMD. If both eyes presented simultaneously, right eye was designated as study eye.

### Outcomes

The primary outcome was the number of patients forecasted to require initiation of anti-VEGF therapy during the UK national lockdown between 16 March 2020 and 31 July 2020. Predicted visual outcomes if patients were left untreated were also assessed, including: change in VA from baseline; VA of ≥70 ETDRS letters (Snellen equivalent 6/12); VA of <25 ETDRS letters (6/96) and VA of ≤20 ETDRS letters (3/60). Each of these end points were considered over 12 months following predicted treatment initiation.

The threshold of 70 ETDRS letters (6/12) is an indicator of patient independence and thus commonly used to signify positive visual outcomes. It is: (1) the International Council of Ophthalmology’s threshold for good independent vision^17 18^; (2) the legal threshold for driving in the UK^19^ and (3) the minimum VA required to read small print.^20^ Accordingly, it is recommended as a key threshold by numerous consortia.^17 18^ Conversely, a VA of <25 ETDRS letters (6/96) indicates poor visual prognosis as it precludes an eye from treatment with anti-VEGF.^15^ Similarly, a VA of ≤20 ETDRS letters (3/60) is the criterion for severe sight impairment registration in the UK.

All data analyses were carried out with R (V.3.5.1).^21^ Calculated means in text and figures are expressed with ±SD, unless otherwise specified.

### Forecasting nAMD patients

Historical data from 2018 and 2019 for the number of patients initiating anti-VEGF therapy for nAMD by calendar week were used as the basis for a forecast predicting the number of patients needing to initiate anti-VEGF therapy for nAMD during the national lockdown. The number of patients needing to initiate anti-VEGF therapy for nAMD during a period of UK national lockdown between 16 March 2020 and 31 July 2020 (weeks 12 through 31) was forecasted using an additive model consisting of a trend component, a seasonal component and a random component alongside Holt Winters exponential smoothing.^22 23^

### Sampling hypothetical nAMD patients based on forecast

Hypothetical patient profiles were derived for the patients forecasted to require initiation of anti-VEGF therapy for nAMD during the national lockdown. Specifically, hypothetical patients were randomly sampled from patients attending MEH and UHB during the same time period (16 March to 31 July) in 2018 and 2019.

### Propensity scoring and matching of forecasted patients to MARINA trial sham-control patients

Logistic regression was used to generate a propensity score for each patient in the hypothetical cohort of patients needing to initiate anti-VEGF therapy for nAMD during the national lockdown from the following patient characteristics: age, sex and VA. Using a calliper width of 0.1 of the SD of the logit of the propensity score, the hypothetical patients were matched to corresponding MARINA sham-control arm patients.

### Modelling visual outcomes for non-matched patients

For MEH and UHB patients that could be matched exactly to MARINA patients based on age, sex and VA (matched patients), VA values over time were described using the MARINA sham-control arm matched patient data. For MEH and UHB patients with characteristics out with the MARINA inclusion criteria (non-matched patients), VA was predicted using a simple linear model based on MARINA trial patients with patient characteristics such as age, sex and VA as covariates.

## Results

### Cohort characteristics at treatment initiation

Between 1 January 2018 and 15 March 2020, 1929 treatment-naïve patients (60.5% female) initiated anti-VEGF therapy for nAMD at MEH and 267 patients (68.5% female) at UHB (table 1A). Most were 65 years or older, with only 6.5% and 3.0% of patients under 65 at MEH and UHB, respectively. The mean VA of eyes initiating anti-VEGF therapy for nAMD between 1 January 2018 and 15 March 2020 at MEH was 55.9±16.3 ETDRS letters (Snellen equivalent 20/80±1 line), slightly higher than at UHB where it was 51.7±20.0 ETDRS letters (20/100±1 line (two sample t-test p<0.05; table 1B and online supplemental figure 1). Patient cohorts treated at MEH and UHB were analysed separately given observed differences in demography and clinical features at treatment initiation (table 1).

10.1136/bjophthalmol-2021-319383.supp3 Supplementary data

**Table 1:** Cohort demography and clinical features at baseline

	Overall (n=2196)	MEH (n=1929)	UHB (n=267)
**A**			
Sex n (%)			
Female	1350 (61.5)	1167 (60.5)	183 (68.5)
Male	846 (38.5)	762 (39.5)	84 (31.5)
Age (years) n (%)			
<65	133 (6.1)	125 (6.5)	8 (3.0)
65–69	154 (7.0)	139 (7.2)	15 (5.6)
70–74	312 (14.2)	271 (14.0)	41 (15.4)
75–79	407 (18.5)	369 (19.1)	38 (14.2)
80–84	515 (23.5)	447 (23.2)	68 (25.5)
85+	675 (30.7)	578 (30.0)	97 (36.3)
Ethnicity n (%)			
White	1104 (50.3)	905 (46.9)	199 (74.5)
Not white	959 (43.7)	945 (49.0)	14 (5.2)
Not reported	133 (6.1)	79 (4.1)	54 (20.2)
Smoking status n (%)			
Yes	149 (6.8)	140 (7.3)	9 (3.4)
**B**			
Visual acuity (VA) ETDRS letters			
Mean (SD)	55.4 (16.8)	55.9 (16.3)	51.7 (20.0)
Median (min, max)	59.0 (0, 94.0)	60.0 (0, 90.0)	55.0 (0, 94.0)
VA ≥70 n (%)			
Yes	563 (25.6)	503 (26.1)	60 (22.5)
No	1633 (74.4)	1426 (73.9)	207 (77.5%)
VA ≤20 n (%)			
Yes	55 (2.5)	40 (2.1)	15 (5.6)
No	2141 (97.5)	1889 (97.9)	252 (94.4)
VA <25 n (%)			
Yes	139 (6.3)	112 (5.8)	27 (10.1)
No	2057 (93.7)	1817 (94.2)	240 (89.9)
No or unknown	2047 (93.2)	1789 (92.7)	258 (96.6)

Mean, SD and distribution are shown for (A) demography (sex, ethnicity, smoking status and age) and (B) visual parameters in approximate ETDRS letters of patients with nAMD at initiation of anti-VEGF therapy at MEH and UHB.

MEH, Moorfields Eye Hospital; nAMD, neovascular age-related macular degeneration; UHB, University Hospitals Birmingham; VEGF, vascular endothelial growth factor.

### Forecasting patients needing to initiate anti-VEGF therapy for nAMD during UK national lockdown

**Figure 1:**
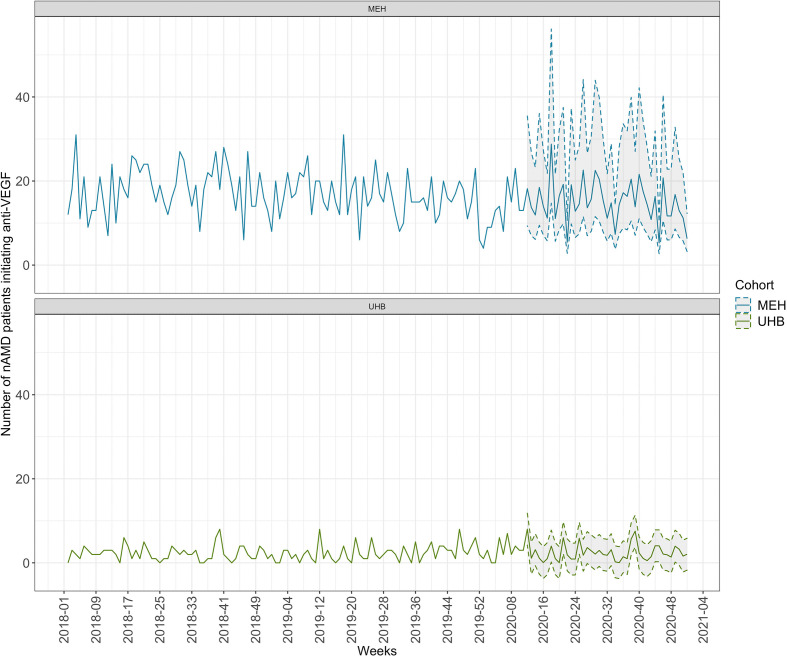
Period trends of anti-vascular endothelial growth factor (anti-VEGF) treatment initiation for neovascular age-related macular degeneration (nAMD). Actual (solid line) and predicted (solid line with CIs) number of patients per week needing to initiate anti-VEGF therapy for nAMD at Moorfields Eye Hospital (MEH) (top panel; blue) and University Hospitals Birmingham (UHB) (bottom panel; green); 95% (grey) CI are depicted.

Our model to forecast the number of patients needing to initiate anti-VEGF therapy for nAMD during a period of UK national lockdown between 16 March 2020 and 31 July 2020 (weeks 12 through 31) predicted a total of 325 patients in need of initiating anti-VEGF treatment over 20 weeks at MEH (figure 1 and online supplemental table 1A). At UHB, 51 nAMD patients were forecasted for initiation of anti-VEGF treatment in that period (figure 1 and online supplemental table 1B).

10.1136/bjophthalmol-2021-319383.supp1 Supplementary data

### Predicted visual acuity changes in the absence of anti-VEGF therapy

**Figure 2:**
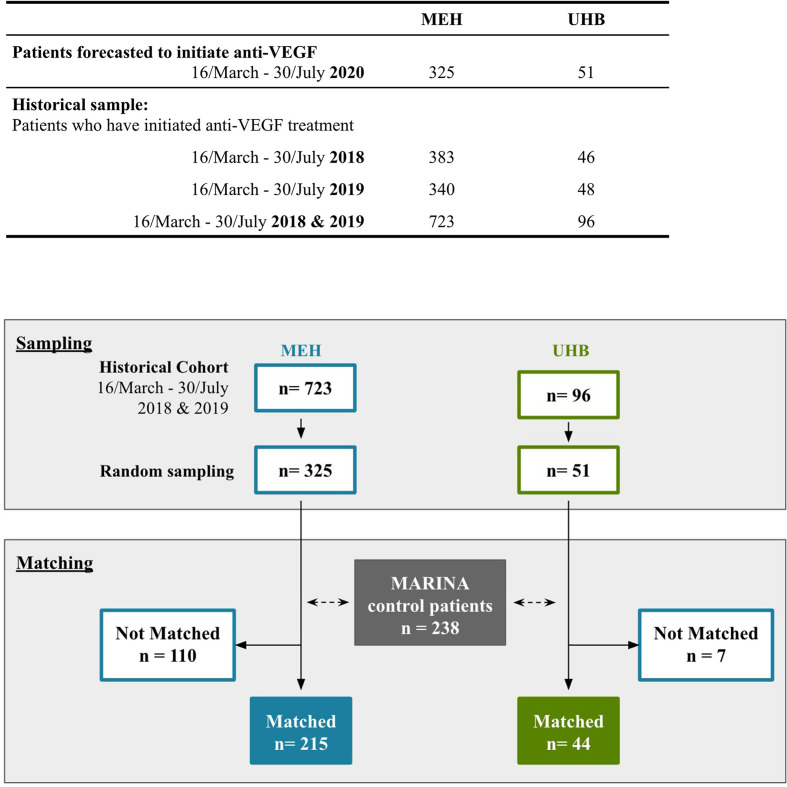
Sampling and matching process to generate cohorts for predicting visual acuity (VA) changes. Flow diagram outlining how patient profiles were generated to model VA changes during a national lockdown. In brief, profiles for the number of neovascular age-related macular degeneration (nAMD) patients forecasted to initiate anti-vascular endothelial growth factor (anti-VEGF) therapy between 16 March 2020 and 31 July 2020 were randomly selected from the pool of patients who initiated therapy in the same period in the two preceding years at Moorfields Eye Hospital (MEH) and University Hospitals Birmingham (UHB). These profiles were matched to patients in the sham-control arm of the Minimally Classic/Occult Trial of the Anti-VEGF Antibody Ranibizumab in the Treatment of Neovascular AMD (MARINA) study on sex, age, VA, VA ≥70 and VA ≤20 at treatment initiation. Some MEH and UHB patients could not be matched to MARINA patient profiles, due to the trial exclusion criteria. Predicted changes in these patients were based on a linear model of VA changes developed from the trial population and extrapolated to the unmatched population. The number of matched and unmatched patients for both NHS Trusts is shown.

Next, the impact of absent anti-VEGF treatment due to a national lockdown on vision in nAMD patients was modelled. Here, patients that had presented to MEH and UHB within the same 20-week time period in the 2 years preceding the national lockdown in the UK were randomly selected (figure 2). These patient profiles were matched on demography and clinical features with participants receiving sham-treatments as part of the sham-control arm of the MARINA study,^8^ the last study to include non-VEGF-treated patients.

The resultant cohort comprised 215 matched and 110 unmatched patient profiles for MEH (UHB: 44 matched and 7 unmatched). Table 2 summarises the presenting characteristics for the patients in the MARINA sham arm, as well as, matched and unmatched MEH and UHB cohorts. A smaller proportion of MEH patients matched to patient profiles in the sham-control arm of the MARINA trial when compared with UHB.

**Table 2:** Demography and clinical features at anti-VEGF initiation for the model population

	MARINA sham-arm (n=238)	MEH matched (n=215)	MEH not matched (n=110)	UHB matched (n=44)	UHB not matched (n=7)
Patient demography					
Sex n (%)					
Female	159 (66.8)	128 (59.5)	73 (66.4)	32 (72.7)	7 (100.0)
Male	79 (33.2)	87 (40.5)	37 (33.6)	12 (27.3)	0 (0.0)
Age (years) n (%)					
<65	11 (4.6)	9 (4.2)	6 (5.5)	2 (4.5)	0 (0.0)
65–69	17 (7.1)	19 (8.8)	4 (3.6)	1 (2.3)	0 (0.0)
70–74	50 (21.0)	37 (17.2)	14 (12.7)	4 (9.1)	0 (0.0)
75~79	68 (28.6)	58 (27.0)	13 (11.8)	4 (9.1)	0 (0.0)
80~84	64 (26.9)	60 (27.9)	20 (18.2)	19 (43.2)	0 (0.0)
85+	26 (10.9)	32 (14.9)	53 (48.2)	14 (31.8)	7 (100.0)
Ethnicity n (%)					
White	231 (97.1)	97 (45.1)	55 (50.0)	39 (88.6)	6 (85.7)
Not white	7 (2.9)	118 (54.9)	55 (50.0)	5 (11.4)	1 (14.3)
Smoking status n (%)					
Yes	130 (54.6)	110 (51.2)	55 (50.0)	33 (75.0)	3 (42.9)
No or unknown	108 (45.4)	105 (48.8)	55 (50.0)	11 (25.0)	4 (57.1)
Clinical features					
Visual acuity (VA) (ETDRS letters)					
Mean (SD)	53.61 (14.06)	52.92 (14.45)	59.50 (18.36)	52.55 (15.98)	40.57 (20.98)
Median (min, max)	56.0 (3.0, 84.0)	55.0 (0, 86.0)	70.0 (11.0, 85.0)	55.0 (0, 76.0)	35.0 (11.0, 80.0)
VA ≥70 ETDRS letters n (%)					
Yes	22 (9.2)	16 (7.4)	61 (55.5)	8 (18.2)	1 (14.3)
No	216 (90.8)	199 (92.6)	49 (44.5)	36 (81.8)	6 (85.7)
VA ≤20 ETDRS letters n (%)					
Yes	4 (1.7)	3 (1.4)	3 (2.7)	1 (2.3)	1 (14.3)
No	234 (98.3)	212 (98.6)	107 (97.3)	43 (97.7)	6 (85.7)
VA <25 ETDRS letters n (%)					
Yes	9 (3.8)	12 (5.6)	7 (6.4)	1 (2.3)	1 (14.3)
No	229 (96.2)	203 (94.4)	103 (93.6)	43 (97.7)	6 (85.7)

Patient characteristics and clinical features for the MARINA sham-control cohort as well as the forecasted patients for the matched and non-matched MEH and UHB cohorts are shown.

MARINA, Minimally Classic/Occult Trial of the Anti-VEGF Antibody Ranibizumab in the Treatment of Neovascular AMD; MEH, Moorfields Eye Hospital; UHB, University Hospitals Birmingham; VEGF, vascular endothelial growth factor.

Our data suggest that an interruption to ophthalmic services during a national lockdown period would lead to VA deterioration in new nAMD patients (online supplemental figure 2). If left untreated for 12 months, mean VA was projected to decline from 52.9±14.5 ETDRS letters to 43.6±17.8 ETDRS letters in the MEH-MARINA matched cohort, and from 52.6±16.0 ETDRS letters to 40.6±17.0 ETDRS letters in the UHB-MARINA matched cohort (online supplemental figure 2 and online supplemental table 2). For unmatched patients, VA changes were extrapolated using a separate model, also based on the data from the MARINA sham-control cohort. For the MEH and UHB non-matched patients, mean VA after 12 months was projected to decline from 59.5±18.4 to 41.9±12.3 ETDRS letters and from 40.6±21.0 to 29.5±8.2 ETDRS letters (n=7), respectively (online supplemental table 2). A decline in VA was consistently seen as early as 3 months following failure to initiate treatment.

10.1136/bjophthalmol-2021-319383.supp4 Supplementary data

10.1136/bjophthalmol-2021-319383.supp2 Supplementary data

### Impact on patient quality of life in untreated nAMD due to national lockdown

**Figure 3:**
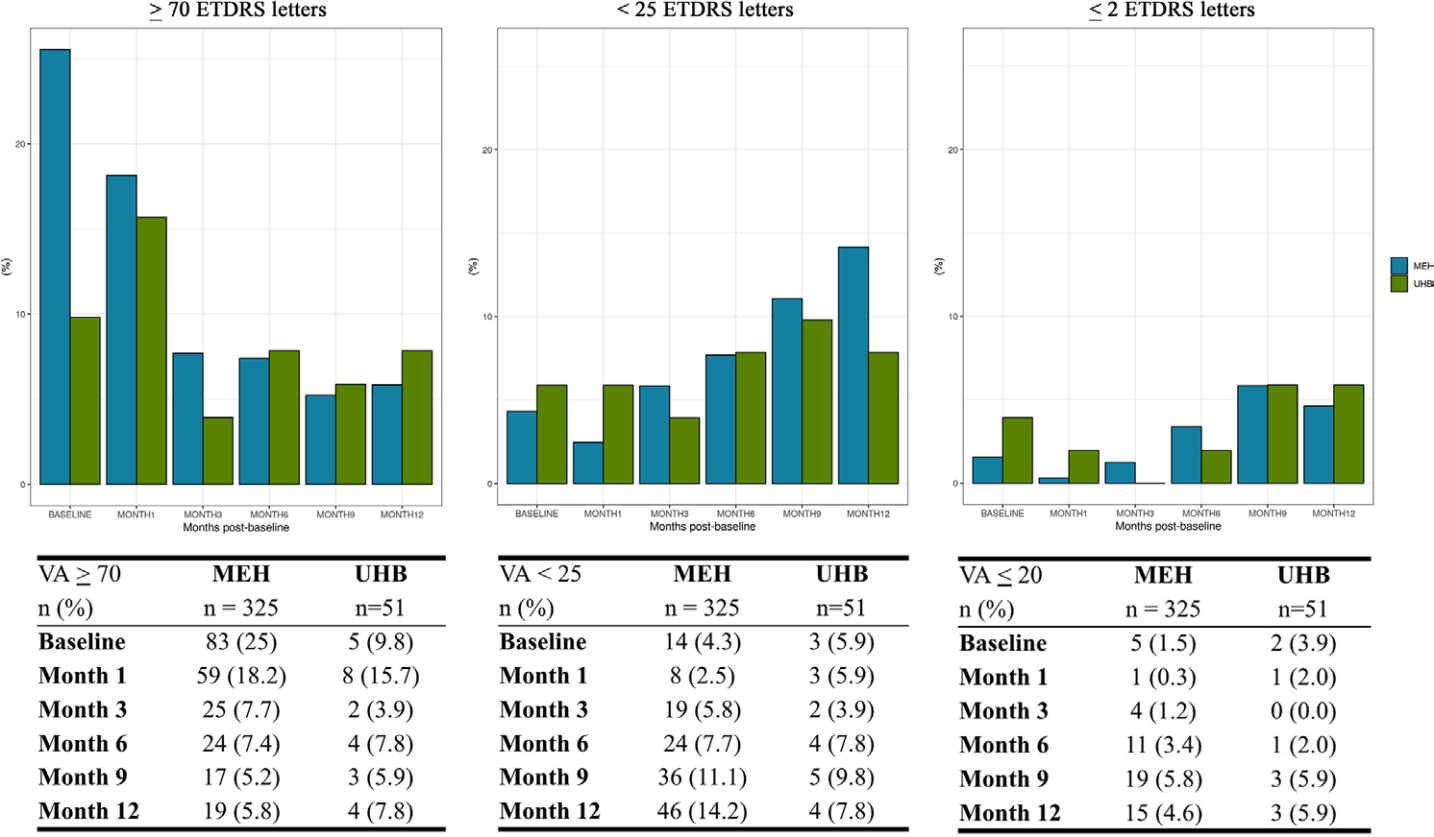
Projected visual acuity (VA) thresholds. Bar charts representing the proportion of study eyes for the Moorfields Eye Hospital (MEH) (blue) and University Hospitals Birmingham (UHB) (green) predicted cohorts over time to (A) achieve a VA of ≥70 ETDRS, (B) fall below a VA of 25 ETDRS letters and (C) have a VA of ≤20 ETDRS letters. Matched and unmatched cohorts were combined for this analysis. Last observation was carried over to keep constant denominators.

Forecasted changes in VA observed here are likely to be clinically significant. At baseline, 25% (83/325) of MEH patients, forecasted to require anti-VEGF, would be expected to have a VA ≥70 ETDRS letters (6/12) in the affected eye, based on a pooled analysis of matched and unmatched cohorts. The pooled UHB cohort was predicted to have a lower VA at baseline where only 9.8% (5/51) of patients with VA ≥70 ETDRS letters (6/12). After 3 months without treatment, this proportion was projected to fall to 7.7% (25/325) and 3.9% (2/51) for MEH and UHB, respectively. After 12 months without treatment, the proportion of eyes with VA ≥70 ETDRS letters (6/12) was projected to be 5.8% (19/325) and 7.8% (4/51) for MEH and UHB, respectively (figure 3A).

For those forecasted to initiate anti-VEGF in the national lockdown period, 14.2% (46/325) and 7.8% (4/51) of study eyes at MEH and UHB, respectively, would not be eligible for treatment (ie, VA <25 ETDRS letters (6/96)) at the 12-month time point without treatment (figure 3B).

Of forecasted nAMD patients, 1.5% (5/325) and 3.9% (2/51) at MEH and UHB cohorts, respectively, were predicted to have a VA of ≤20 ETDRS letters (3/60) at baseline. After 12 months delay in treatment, proportion falling below this threshold is projected to increase to 4.6% (15/325) and 5.9% (3/51) at MEH and UHB, respectively (figure 3C).

## Discussion

### Main findings

Based on data between 2018 and 2019, 376 patients were projected to initiate anti-VEGF therapy for nAMD across MEH and UHB between 16 March 2020 and 31 July 2020. In the absence of anti-VEGF therapy, these patients would have—on average—lost vision. Analyses presented here suggest that a treatment-naïve nAMD patient presenting at the beginning of service disruption (end of April) would have had a reduced chance of retaining good vision (VA ≥70 ETDRS letters (6/12)) once the services resumed at the end of July 2020. Crucially, treatment-delay may also have put them at risk of losing eligibility for treatment, as they may no longer have met the minimum VA requirement of ≥25 ETDRS letters (6/96), in addition to increased risk of severe visual impairment (≤20 ETDRS letters (3/60)). Risks to positive visual outcomes would proportionately increase if service disruption lasted longer than 6 months, either due to: extended lockdown period; reduced capacity on service resumption or if patients did not feel safe to seek medical services.

### Results in the context of the existing literature

Anti-VEGF is used to treat VA deterioration secondary to nAMD.^8 24 25^ Interruption to therapy can negatively impact visual prognosis^26^ and it has been demonstrated that early treatment initiation results in better prognosis for patients with nAMD. Indeed, time-event analyses of MEH nAMD patients demonstrated that probability of attaining 70 ETDRS letters (6/12) increases by 43% for every 5 letters of VA at treatment initiation.^27^ Moreover, a higher baseline VA also increases the duration of retaining positive visual outcome; and treatment delay can have a detrimental effect on vision outcomes.

The vision recovery velocity for anti-VEGF therapy was estimated to be between 0.17 and 0.56 letters per month.^28^ Analyses presented here demonstrated that vision loss could proceed as fast as one letter per month of deferred treatment, that is, at a faster rate than can be regained on a monthly basis with the gold standard treatment. This illustrates the importance of timely treatment initiation for optimising vision outcomes.

The effects of immediate disruption of medical services due to national lockdown is becoming evident, both within AMD pathways,^29^ ophthalmic services^30^ and more broadly across each medical specialty.^31^ Single-centre studies have reported reduction in numbers of newly diagnosed nAMD presenting for treatment between prepandemic and postlockdown time frames, acknowledging the difficulty in predicting treatment outcomes in those for whom treatment was delayed or not initiated.^29^ Retina specialists anticipated potential detrimental effects of delayed diagnosis and interrupted care, and identified ways to mitigate this with accelerated innovation, particularly in healthcare pathway redesign and digitalisation.^32^

### Limitations

Our predictive model for VA over time is based on post hoc analysis of the landmark MARINA trial sham-control arm, which excludes patients with any concurrent intraocular condition in the study eye (eg, cataract or diabetic retinopathy). In the wider population, concurrent other conditions that could impair VA and visual outcomes over time need to be accounted for, and therefore the model may underestimate the loss of VA over time.

A proportion of MEH and UHB patients could not be matched to MARINA patient profiles, due to differences in patient selection for inclusion in the respective cohorts. For example, the majority of patients predicted to fall below 70 ETDRS letters (6/12) are based on patients who did not match the MARINA trial population due to its selection criteria. Predicted changes in these patients were based on a linear model of VA changes developed from the trial population and extrapolated to the unmatched population. Our analyses suggest worse visual outcomes for non-matched patients, especially for the MEH cohort. This effect could reflect the higher mean starting VA in the MEH cohort compared with UHB and MARINA cohorts.

There are two anti-VEGF treatments licensed in the UK, ranibizumab and aflibercept. Aflibercept is most commonly used at MEH, in contrast to ranibizumab at UHB. While both treatments inhibit VEGF signalling in nAMD, any influence of treatment choice on visual outcomes could not be addressed in the present study.

### Implications for further research and practice

This study demonstrates the value of coordinated health data research initiatives such as INSIGHT. Furthermore, insights from prospectively collected routine clinical patient-level hospital data can be maximised by combining them with additional resources, such as randomised clinical trial data. Going forward, the INSIGHT initiative would also enable validation of our model with observed data collected during the first lockdown, as well as model the effects of subsequent lockdowns in the UK. Such initiatives could help the medical community better understand and potentially mitigate the effects of national lockdowns on other chronic health conditions where early treatment initiation is critical for patient outcomes.

## Conclusion

As COVID-19 cases decrease, hospitals seek to progressively restore non-urgent services in the context of competing demands for limited resources. According to our results, a 3-month delay in treatment initiation could potentially result in measurable detrimental effects on vision. Extending the delay for longer periods of time is predicted to proportionately decrease positive visual outcomes and increase negative ones. It is now essential to support patients accessing care by providing safe environments as recommended by national bodies.^33^ Providing optimal patient care, while minimising the risk of infection, resource planning at ophthalmic care centres must take into account the bolus of patients who were undiagnosed or did not initiate therapy during planned lockdowns. This group of patients should be prioritised and resources allocated appropriately to diagnose and treat these patients as they present for care.

This INSIGHT Health Data Research Hub report has modelled the immediate impact of delayed diagnosis of nAMD and estimated the potential of permanent visual morbidity caused by anti-VEGF treatment interruptions secondary to national lockdown. The directed use of MARINA trial data enabled a forecast of this serious morbidity.

## Data Availability

Data may be obtained from a third party and are not publicly available. INSIGHT is a National Health Service-led partnership established to improve healthcare by encouraging research using routinely collected eye data. INSIGHT is funded by Health Data Research, United Kingdom (HDR UK).
