# Supplementary figures and images for: Predicting the immediate impact of national lockdown on neovascular age-related macular degeneration and associated visual morbidity: an INSIGHT Health Data Research Hub for Eye Health report
Source: Br J Ophthalmol. 2021 Sep 13;107(2):267–74. doi: 10.1136/bjophthalmol-2021-319383 (PMC9887382; doi:10.1136/bjophthalmol-2021-319383)

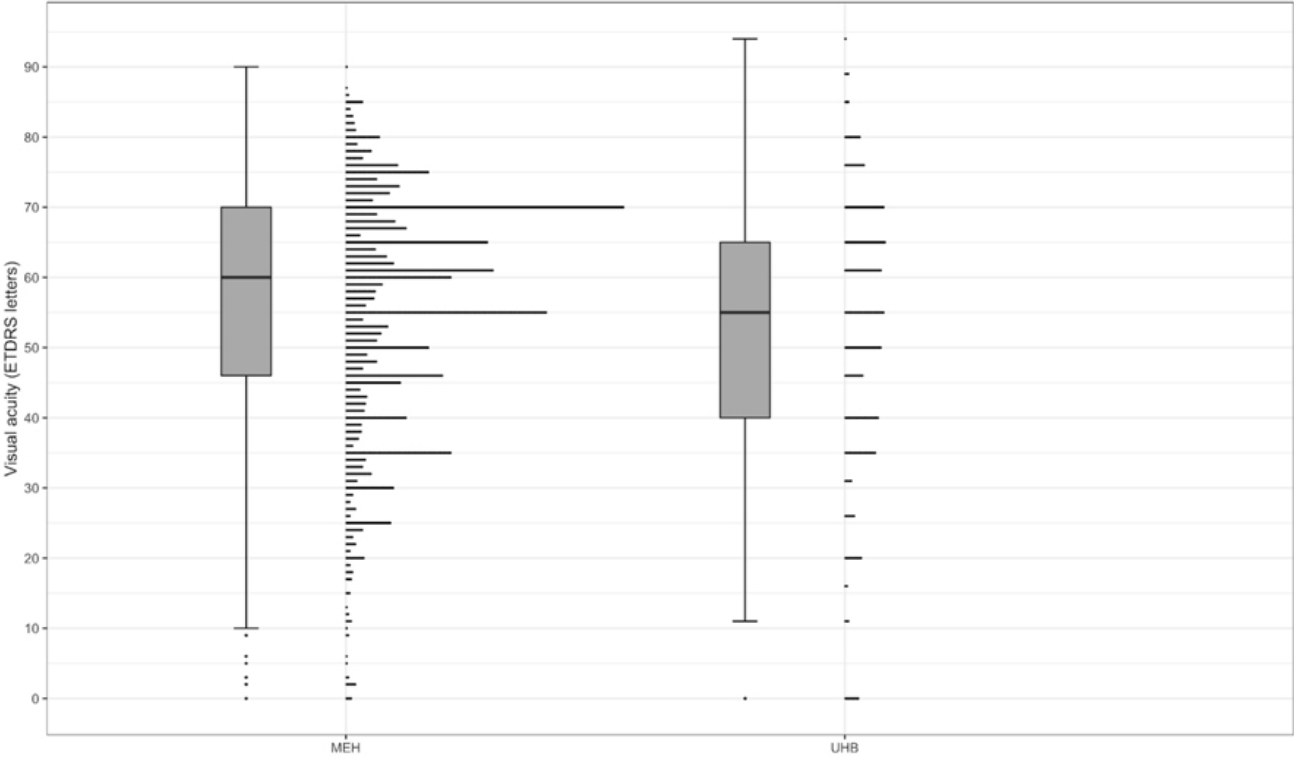

Supplement: Supplementary data [file bjophthalmol-2021-319383supp003.pdf]

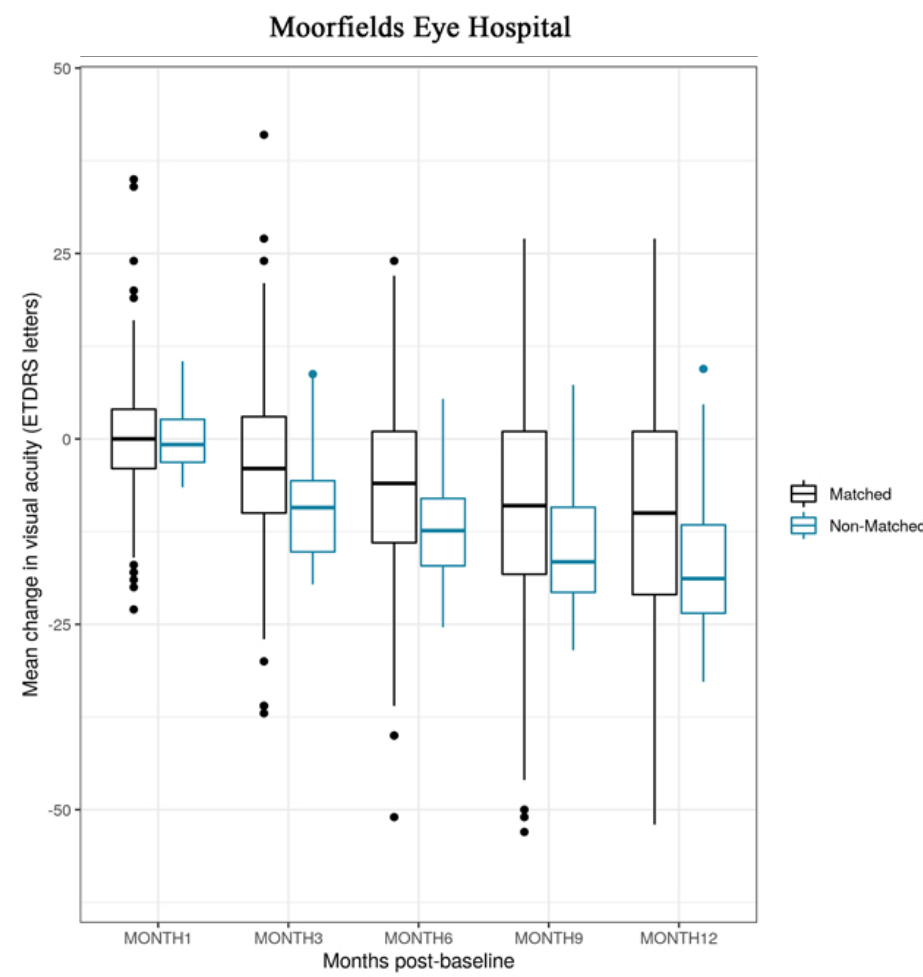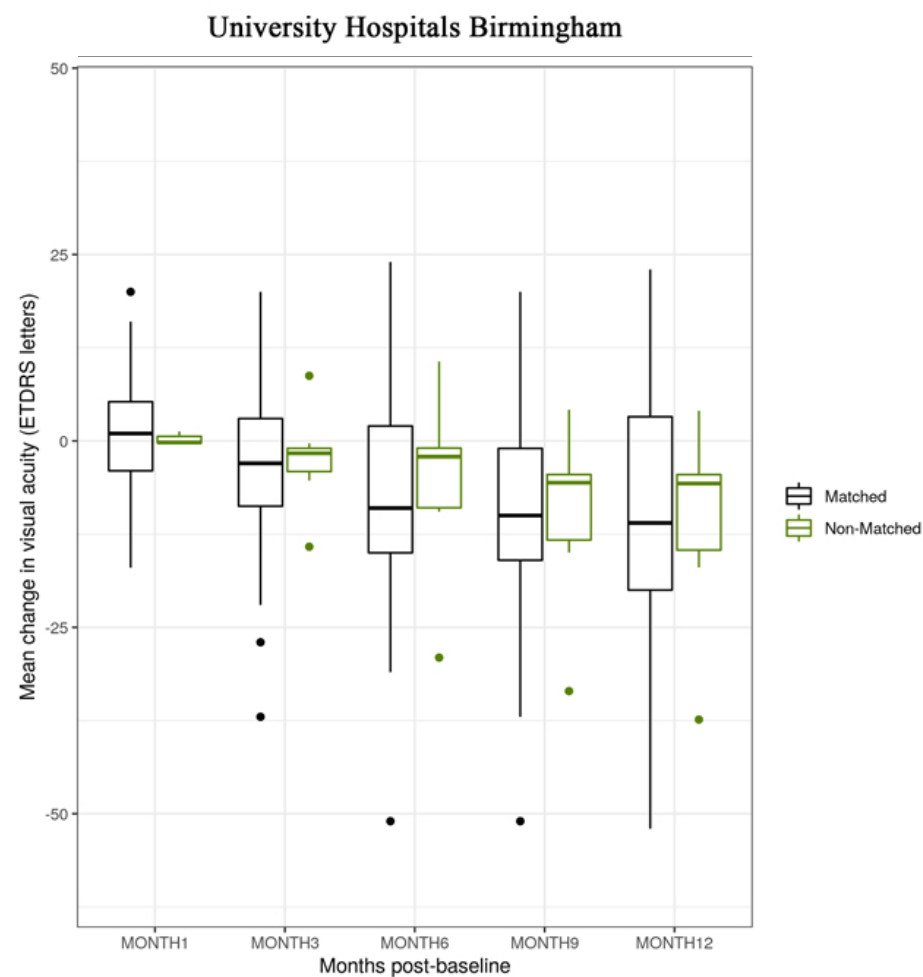

Supplement: Supplementary data [file bjophthalmol-2021-319383supp004.pdf]
