# Supplementary material for: Predicting the immediate impact of national lockdown on neovascular age-related macular degeneration and associated visual morbidity: an INSIGHT Health Data Research Hub for Eye Health report
Source: Br J Ophthalmol. 2021 Sep 13;107(2):267–74. doi: 10.1136/bjophthalmol-2021-319383 (PMC9887382; doi:10.1136/bjophthalmol-2021-319383)
Supplement: Supplementary data [file bjophthalmol-2021-319383supp001.pdf]

a

			Forecasted	
**Week**	**2018**	**2019**	**2020**	**95% CI**
12	24	20	18	(9.3 - 35)
13	10	13	14	(7 - 27)
14	21	12	12	(6.1 - 23)
15	17	20	18	(9.5 - 36)
16	16	15	14	(7.2 - 27)
17	26	12	11	(5.7 - 22)
18	25	31	29	(15 - 56)
19	22	12	11	(5.7 - 22)
20	24	18	16	(8.4 - 32)
21	23	21	19	(9.8 - 38)
22	18	6	5.4	(2.8 - 11)
23	15	21	19	(9.8 - 37)
24	19	14	13	(6.6 - 25)
25	15	16	15	(7.4 - 28)
26	12	24	23	(12 - 44)
27	16	17	14	(7 - 27)
28	19	15	16	(8 - 31)
29	27	21	23	(12 - 44)
30	25	17	20	(10 - 40)
31	19	12	15	(7.9 - 30)
**Total**	**393**	**337**	**325.4**	

b				
			Forecasted	
**Week**	**2018**	**2019**	**2020**	**95% CI**
12	3	8	8.0	(-2.7 - 4.9)
13	2	1	1.1	(-3.7 - 3.9)
14	0	3	3.1	(-2.8 - 4.8)
15	6	1	1.1	(0.2 - 7.8)
16	4	0	0.1	(-2.8 - 4.8)
17	1	1	1.0	(-3.8 - 3.8)
18	3	4	4.0	(2.2 - 9.8)
19	1	1	1.0	(-1.9 - 5.7)
20	5	0	0.0	(-2.9 - 4.7)
21	3	6	6.0	(-2.9 - 4.7)
22	1	2	1.9	(2.1 - 9.7)
23	1	1	0.9	(-2 - 5.6)
24	0	1	0.9	(-0.15 - 7.4)
25	1	6	5.9	(-0.92 - 6.7)
26	1	2	1.8	(-1.7 - 5.9)
27	4	1	3.6	(-0.83 - 6.8)
28	3	2	2.9	(-1.8 - 5.8)
29	2	3	2.1	(-2.7 - 4.9)
30	3	3	3.0	(-3.7 - 3.9)
31	2	2	2.0	(-2.8 - 4.8)
**Total**	**46**	**48**	**50.4**	

Supplementary Table 1. Number of nAMD patients initiating anti-VEGF therapy per week. (a) Moorfields Eye Hospital and (b) University Hospitals Birmingham. Displayed are data for the pre-COVID-19 era and predicted patients in corresponding weeks during the national lockdown period with 95% confidence level for prediction intervals.

Mollan SP, *et al. Br J Ophthalmol* 2023; 107:267–274. doi: 10.1136/bjophthalmol-2021-319383
